# Diagnostic value of anti-human citrullinated fibrinogen ELISA and comparison with four other anti-citrullinated protein assays

**DOI:** 10.1186/ar2011

**Published:** 2006-07-19

**Authors:** Bert Vander Cruyssen, Tineke Cantaert, Leonor Nogueira, Cyril Clavel, Leen De Rycke, Amélie Dendoven, Mireille Sebag, Dieter Deforce, Christian Vincent, Dirk Elewaut, Guy Serre, Filip De Keyser

**Affiliations:** 1 Department of Rheumatology, Ghent University Hospital, Ghent, Belgium; 2 UMR 5165 'Laboratory of Epidermis Differentiation and Rheumatoid Autoimmunity', CNRS – Toulouse III University, Toulouse, France; 3 Laboratory of Pharmaceutical Biotechnology, Ghent University, Ghent, Belgium

## Abstract

We studied the diagnostic performance of the anti-human citrullinated fibrinogen antibody (AhFibA) ELISA for rheumatoid arthritis (RA) in a consecutive cohort (population 1) and evaluated the agreement between the AhFibA ELISA and four other assays for anti-citrullinated protein/peptide antibodies (ACPAs) as well as rheumatoid factor in patients with longstanding RA (population 2). Population 1 consisted of 1024 patients with rheumatic symptoms; serum samples from these patients were sent to our laboratory for ACPA testing within the context of a diagnostic investigation for RA. Ninety-two of these patients were classified as having RA according to the American College of Rheumatology criteria and 463 were classified as non-RA patients. Population 2 consisted of 180 patients with longstanding RA and was used to assess agreement and correlations between five ACPA assays: anti-cyclic citrullinated peptide (CCP)1 and anti-CCP2 antibodies were detected using a commercially available ELISA, AhFibA using ELISA, and anti-PepA and anti-PepB antibodies using line immunoassay. Applying previously proposed cut-offs for AhFibA, we obtained a sensitivity of 60.9% and a specificity of 98.7% in population 1. Receiver operating characteristic curve analysis could not detect a significant difference in diagnostic performance between the AhFibA ELISA and anti-CCP2 assay. Performing a hierarchical nearest neighborhood cluster analysis of the five different ACPA assays in population 2, we identified two clusters: a cluster of anti-pepA, anti-pepB and anti-CCP1, and a cluster of AhFibA and anti-CCP2. In conclusion, we found that AhFibA and anti-CCP2 antibodies had similar diagnostic performance. However, disagreement between ACPA tests may occur.

## Introduction

Clinical indicators of rheumatoid arthritis (RA) are pain and swelling of the proximal interphalangeal and metacarpophalangeal joints. Larger joints such as knee, elbow and ankle joints may also be affected. Synovial inflammation and joint destruction together with the extra-articular manifestations of the disease are responsible for a severe decline in the RA patient's quality of life. It is important to identify RA early. Joint erosions, which are irreversible, occur early in the disease process and intervention with aggressive therapy is most successful if it is applied early in the disease course [1]. A sensitive and specific serological test is needed for application in this window in the disease course, when often not all clinical manifestations are apparent.

Several autoantibody systems have been described in this autoimmune disease [2]. The presence of the rheumatoid factor (RF), directed against the Fc part of an IgG molecule, is one of the American College of Rheumatology (ACR) criteria for RA [3]. This antibody is present in about 65–75% of RA patients. However, because it is also found in patients with other autoimmune diseases or infectious diseases, and even in the healthy elderly, it has limited specificity. The presence of anti-citrullinated protein/peptide antibodies (ACPAs), on the other hand, is significantly more specific for RA. ACPAs are directed against various proteins that have one trait in common; some of their arginines have been converted to citrulline by post-translational modification, catalyzed by peptidylarginine deiminase enzymes [4,5].

Depending on the substrate, various assays for detection of ACPAs have been developed. Human buccal mucosa cells and rat oesophagus provide the antigenic substrate for anti-perinuclear factor and anti-keratin antibodies [6,7]. The difficulty in standardizing these natural substrates, together with arbitrary interpretation of the indirect immunofluorescence pattern, has hampered the widespread use of these tests. Because it was shown that both anti-perinuclear factor and anti-keratin antibodies reacted against citrullinated filaggrin related proteins [8], the latter was used for detection of ACPAs in immunoblot assays and in an ELISA, resulting in an assay with 52% sensitivity at a specificity of 95% in a cohort of patients with established disease [9,10]. An ELISA using a cyclic citrullinated peptide (CCP) derived from filaggrin was commercialized (anti-CCP1) [11]. Numerous studies were reported in which sensitivities ranged from 41% (with a corresponding specificity of 97.8% [12]) to 68% (with a corresponding specificity of 98% [11]) in established RA. The line immunoassay format was used with two filaggrin based peptides (pepA and pepB), obtained from the results of epitope mapping as well as molecular modelling and computational chemistry [13]. The sensitivity of this assay was 63.6% for pepA and 54.2 % for pepB at a specificity of 98.5% in established RA [14]. With the development of the second generation anti-CCP2 ELISA, sensitivities ranging from 65% [15] to 80% [16] at a high level of specificity have been reported in established RA. Recently, the presence of citrullinated fibrin in the synovial membrane of RA patients [17] and the use of citrullinated fibrinogen to assay the serum antibodies to deiminated fibrinogen (anti-human citrullinated fibrinogen antibody [AhFibA]) was described [18,19].

The aim of the present study was to assess the diagnostic performance of the AhFibA ELISA for RA in a consecutive population of patients of whom serum was sent to our laboratory for RA serological testing. We also studied the agreement between five different ACPA assays and RF in a cohort of patients with established RA.

## Materials and methods

### Study population 1

Study population 1 was established to evaluate the diagnostic performance of the AhFibA assay and to compare the diagnostic performance with an anti-CCP2 antibody assay by means of receiver operator characteristic (ROC) curve analysis. This cohort consisted of 1024 patients with rheumatic symptoms, from whom serum samples were consecutively sent to our laboratory for ACPA determination within the context of a diagnostic investigation. Patients were diagnosed by a clinician by reviewing of files and the patients were classified in accordance with the ACR classification criteria for RA [3]. Eighty-one patients were lost to follow up. We thus diagnosed 92 individuals as having RA, and all of these patients met the ACR criteria for RA. In 463 patients the diagnosis of RA could be excluded. A further 388 patients had undifferentiated arthritis and were further withdrawn from the analysis. The most frequent diseases diagnosed in the non-RA patients were osteoarthritis (31%), soft tissue mechanical complaints 20% (including peri-arthritis scapulohumeralis and tendinopathies), spondyloarthropathy (13%), systemic lupus erythematosus (9%), vasculitis (6%), polymyalgia rheumatica (5%), other connective tissue diseases (including scleroderma and Sjögren's syndrome; 2%), adult patients with juvenile idiopathyic arthritis (1%), psoriatic arthritis (5%), crystal arthritis (3%) and other diseases including infections, malignancies and neurological disorders (5%).

Of the RA patients, 65.2% were female, the median age was 55 years (range 22–85 years) and the median disease duration was 3 years (range 0–40 years). In the non-RA patients, 66.2% were female and the median age was 51 years (range 11–83 years). Of the RA patients 64% were receiving disease-modifying antirheumatic drug therapy, predominantly methotrexate (*n *= 34), sulphasalazine (*n *= 11) and leflunomide (*n *= 5). Combination therapies were administered to six patients. None of the patients were being treated with anti-tumour necrosis factor therapy at the time of sampling. Corticosteroids were being received by 30% of patients.

### Study population 2

Study population 2 consisted of 180 consecutive RA patients with longstanding disease of at least 4 years (median disease duration 9 years; range 4–39 years) [14]. In this cohort we compared the AhFibA assay with four other ACPA assays. All patients were treated with classic disease-modifying antirheumatic drug therapy (methotrexate, gold salts, or sulphasalazine). None of the patients received leflunomide, anti-tumour necrosis factor therapy, or other biologicals. Concomitant corticosteroids were used in one-third of the patients.

### Rheumatoid factor assay

RF was determined using the latex fixation test. A suspension of uniform polystyrene particles sensitized in glycine buffer with heat-altered human IgG (Difco Laboratories, Detroit, MI, USA) was diluted 1/20 and incubated with progressive dilutions of human sera in microtitre wells. The reagents were mixed and incubated at 37°C for 2 hours. The plates were then shaken gently and inspected for observable agglutination. The dilution titre present in the last well showing agglutination was recorded.

### Detection of anti-pepA and anti-pepB antibodies by line immunoassay

Anti-pepA and anti-pepB Abs were detected by a research line immunoassay containing two citrulline-containing peptides, as described previously (INNO-LIA™ RA [for research use only]; Innogenetics, Ghent, Belgium) [13,14]. The cut-off defined for anti-pepA and anti-pepB antibodies corresponds with a specificity of 100% and 99.3% and a sensitivity of 63.6% and 54.2%, respectively [14].

### Detection of anti-CCP1 and anti-CCP2 antibodies by ELISA

Anti-CCP1 and anti-CCP2 antibodies were detected using a commercially available ELISA containing synthetic CCPs (Immunoscan RA, mark 1 and mark 2; Eurodiagnostica, Arnhem, The Netherlands). The ELISA was performed in accordance with the manufacturer's instructions. Briefly, serum samples were diluted 1/50 with dilution buffer and incubated for 1 hour at 37°C. After removing the liquid and washing three times with rinsing buffer, the conjugate solution (peroxidase conjugated anti-human IgG antibodies) was added into each well and incubated for 1 hour at 37°C. After three washing steps with rinsing buffer, the substrate solution (tetramethyl benzidine) was added and incubated for 30 min at room temperature. The stop solution (sulfuric acid; 0.5 mol/l) was added and the absorbance values were read immediately at 450 nm. A 98.5% specificity cut-off has previously been set at 42 U/ml (sensitivity 75.4%) for the anti-CCP2 assay [14] and a 98% specific cut-off has been set at 92 U/ml for the anti-CCP1 assay [11].

### Detection of AhFibA antibodies by ELISA

The AhFibA-ELISA was developed previously [18,20]. Briefly, plasminogen depleted human fibrinogen (Calbiochem, Meudon, France) was further affinity purified on a protein G column (HiTrap protein G; Amersham Biosciences, Orsay, France). Deimination was performed for 2 hours at 37°C with 7 units rabbit skeletal muscle peptidylarginine deiminase per milligram fibrinogen (Sigma, Lyon, France) in deimination buffer (0.1 mol/l Tris-Hcl [pH 7.4], 10 mmol/l CaCl_2_, 5 mmol/l DTT). Microtitration plates (MaxiSorp, Nunc, Denmark) were coated overnight with human deiminated fibrinogen (5 μg/ml) diluted in phosphate-buffered saline (PBS; pH 7.4). The plates were blocked with PBS containing 2% bovine serum albumin. A volume of 100 μl sera, diluted to 1:50 in 2 mol/l NaCl PBS, was applied and plates were incubated for 1 hour. After washing, plates were incubated with horseradish peroxidase labelled goat anti-human IgG antibodies (γ-chain specific) for 1 hour and washed again. All incubations and washing steps were performed at 4°C. Bound antibodies were detected with ortho-phenylene diamine dihydrochloride (Sigma, St. Louis, MO, USA). The reaction was stopped with 50 μm of 3 mol/l sulphuric acid.

Plates were read using a Multiskan plate reader (Thermo Labsystem, Cergy-Pontoise, France). Serum samples were tested twice and results were averaged. A serum was considered positive for AhFibA above a previously defined cut-off corresponding with the 98.5% specificity level (optical densitiy (OD) ≥ 0.12 nm) [18].

### Statistical analysis

ROC curve analyses were performed. Differences between areas under the curve were evaluated, as proposed by Hanley [21]. Agreement between dichotomized variables was measured by the (weighted) κ statistic. Proportions of matched-pair data were compared by means of the McNemar test. A hierarchical nearest neighbourhood cluster analysis of variables was performed based on squared Euclidian distances. All analyses were performed using the commercial available statistical package SPSS 11.0 (SPSS Institute Inc., Chicago, Il, USA).

## Results

### Diagnostic performance of the AhFibA assay for RA and comparison with the anti-CCP2 and RF assay in population 1

The previously proposed AhFibA cut-off of OD ≥ 0.12 corresponded to a specificity level of 98.5% [18]. When we applied this cut-off to population 1, we obtained a sensitivity of 60.9% and a specificity of 98.7% for the AhFibA assay. Seven non-RA patients tested false positive for AhFibA: one patient with systemic lupus erythematosus, one with psoriatic arthritis, one with polymyositis, one with polymyalgia rheumatica and three with osteoarthritis.

**Figure 1:**
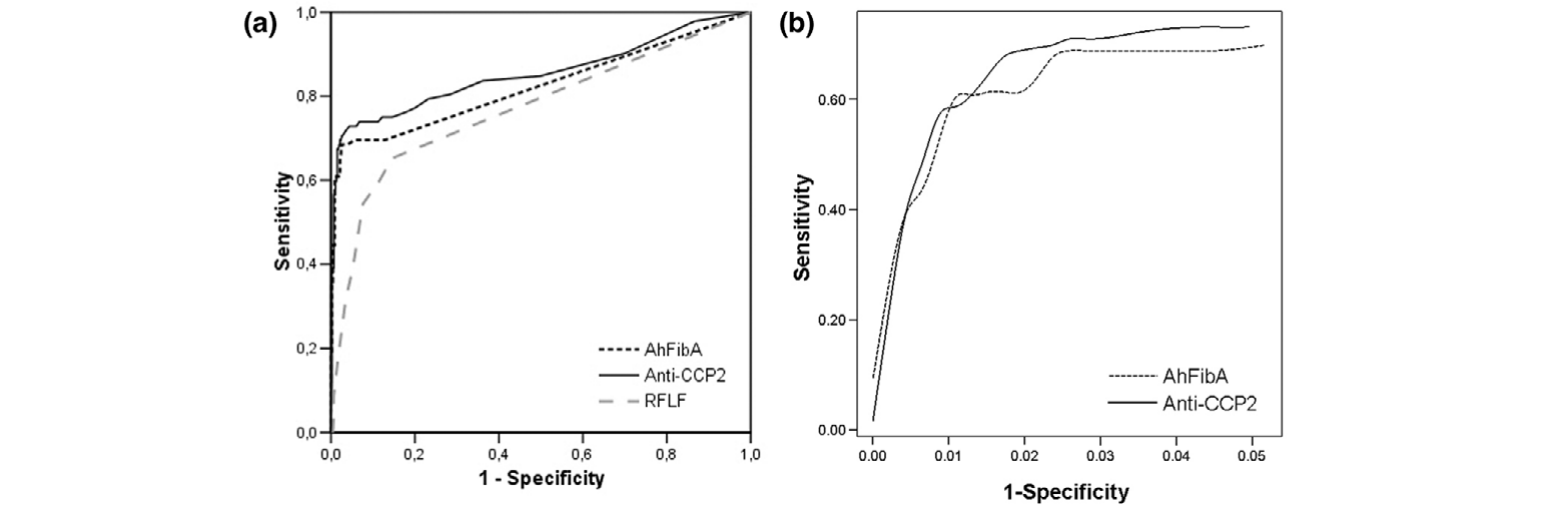
ROC curve analyses of the RF, AhFibA and anti-CCP2 assay in population 1. **(a) **The whole curve is shown, and **(b) **with a focus on detail of the ROC curve at the 98% specificity level for the AhFibA and anti-CCP2 assay in population 1. AhFibA, anti-human fibrinogen (auto)antibodies; CCP, cyclic citrullinated peptide; RF, rheumatoid factor; ROC, receiver operating curve.

The ROC curve comparing the diagnostic performance of AhFibA ELISA, anti-CCP2 assay, and RF in population 1 is shown in Figure 1a, with detail of the curve in the high specificity region shown in Figure 1b. There were no significant differences in the area under the ROC curve analyses of the AhFibA assay compared with the anti-CCP2 assay (0.824 versus 0.854; *P *= NS) [21]. The sensitivities at cut-offs defining comparable specificity levels were similar for the AhFibA ELISA and the anti-CCP2 assay, but they were significantly higher than the sensitivities of the RF test (Table 1). Applying the McNemar test, there were no significant differences between the two ACPA tests after dichotomization at the cut-offs presented in Table 1.

### Agreement between the AhFibA assay and anti-CCP2 in population 1

**Figure 2:**
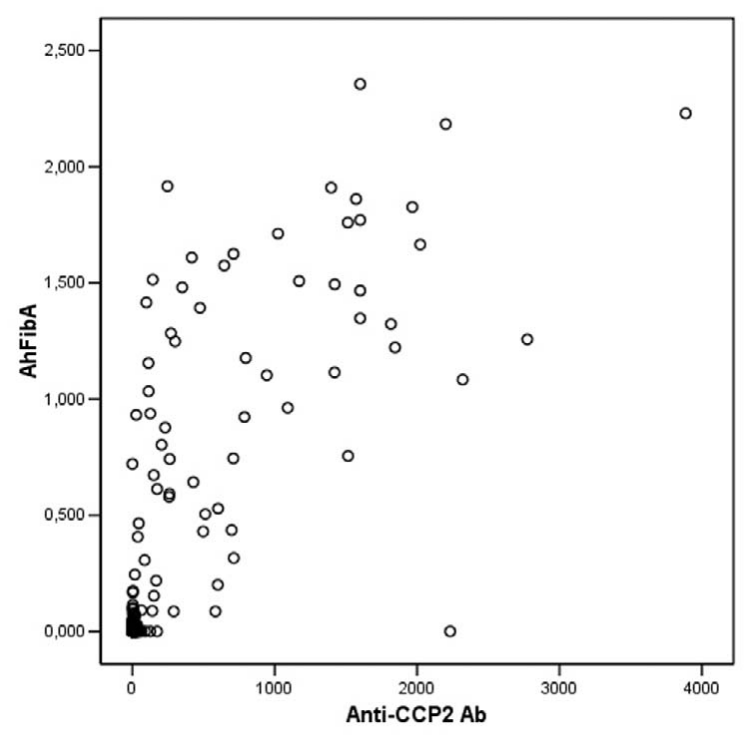
Scatter plot: AhFibA assay versus anti-CCP2 assay in population 1. Ab, antibody; AhFibA = anti-human fibrinogen (auto)antibodies; CCP = cyclic citrullinated peptide.

In Figure 2 the results of the anti-CCP2 assay are plotted against the results of the AhFibA assay. Table 2 shows the cross-tabulation of the results of the AhFibA and anti-CCP2 ELISAs after dichotomization at the 98.5 % specificity level both for the RA and the non-RA patients. The κ statistic, as a measure of agreement between AhFibA and anti-CCP2 ELISA, calculated on the global population, was 0.845. After splitting the population into RA and non-RA patients, we obtained a κ of 0.765 for the RA patients and κ of 0.420 for the non-RA patients. Hence, agreement of both assays is especially impaired in non-RA patients; only 11 non-RA patients exhibited any ACPA reactivity, of which only three were positive for both AhFibA and anti-CCP2 ELISAs. Those three patients had the following diagnoses: osteoarthritis, psoriatic arthritis and polymyositis. We calculated that the specificity in case of double ACPA positivity is 99.4%, with a sensitivity of 58.7%.

### Agreement between the five ACPA assays and RF in population 2

**Figure 3:**
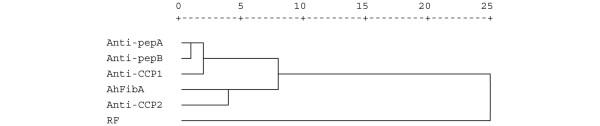
Dendrogram of the cluster analysis of the different ACPA assays in population 2. ACPA, anti-citrullinated protein/peptide antibody; AhFibA, anti-human fibrinogen (auto)antibodies; CCP, cyclic citrullinated peptide; RF, rheumatoid factor.

In this cohort of longstanding RA patients, the agreement between the AhFibA assay and the anti-CCP2 assay corresponded with the agreement observed in the RA patients of population 1. After dichotomization at a >98% specificity level, as defined in Materials and methods (see above), we calculated the sensitivities listed in Table 3; sensitivities, especially for the AhFibA assay, observed in population 2 were higher than those in population 1. The results of the κ statistic as a measure of agreement between dichotomized tests are listed in Table 4, confirming the moderate agreement between the different ACPA tests. A hierarchical nearest neighborhood cluster analysis of variables was performed with the results of RF and five ACPA assays: anti-CCP1, anti-CCP2, anti-pepA, anti-pepB and AhFibA (Figure 3).

This analysis identified the ACPAs apart from the RF. Within ACPAs, we identified different clusters: a cluster of pepA, pepB and anti-CCP1, and a cluster of AhFibA and anti-CCP2.

## Discussion

In the present study, we describe the diagnostic performance of an assay, based on the detection of AhFibA. We compared the diagnostic value of the AhFibA ELISA and the anti-CCP2 ELISA, and conclude that both assays perform equally well, which is reflected by similar ROC curves and similar sensitivities and specificities. There were some non-significant differences in sensitivities of the AhFibA and anti-CCP2 assay between populations 1 and 2.

In contrast to the comparable diagnostic performance of the AhFibA and anti-CCP2 antibodies, the agreement between the two assays in population 1 was only moderate, and was especially impaired in the non-RA patients. Indeed, at the 98.5% specificity level only 11 non-RA patients exhibited any ACPA reactivity, of which only three were positive for both AhFibA and anti-CCP2 antibodies (Table 2). Double ACPA positivity thus resulted in a specificity of 99.4% with a sensitivity of 58.7%.

In population 2, we also evaluated the agreement between the AhFibA ELISA and four other ACPA tests. This confirmed the moderate agreement between the different ACPA assays. Agreement between the different ACPA assays may be important for the implementation of prediction models. Different prediction models for diagnosis of (persistent) erosive disease have been described by means of different ACPA assays [22,23]. Taking into account the similarities between the different ACPA tests, we performed a cluster analysis. Separated from RF, we found a clustering of anti-pepA, anti-pepB and anti-CCP1 assays on one side and anti-CCP2 and AhFibA assays on the other. The clustering of RF at a long distance from ACPAs illustrates the different nature of the antibody systems [24]. Two different explanations can be hypothesized to account for the two clusters within the ACPA tests. First, the anti-pepA, anti-pepB and anti-CCP1 assay use a citrullinated epitope derived from filaggrin. Filaggrin is not the natural autoantigen for ACPA because it is only expressed in epidermis. The substrate of the anti-CCP2 ELISA comprises cyclic peptides selected from libraries containing citrullinated peptides screened with RA sera; these peptides could have a lower degree of homology with filaggrin [25]. The second potential explanation is that both the AhFibA and the anti-CCP2 ELISA use multiple citrullinated epitopes for the detection of ACPAs. Because it was demonstrated that individual RA patients reacted with different citrullinated epitopes [5], the sensitivity of an ACPA test is expected to increase when more than one citrullinated epitope is used.

Increasing the sensitivity at a high specificity level for ACPA detection appears difficult to achieve. Further characterization of the synovial citrullinated proteins apart from fibrinogen may provide new substrates for detection of ACPAs, which might increase the sensitivity and specificity of the future ACPA assays [26]. However, it can be hypothesized that there may be a limit to the sensitivity of ACPA assays for RA. It could be argued that there are two subpopulations within RA patients [27,28]: a population with ACPAs can be detected, which has an increased prevalence of the HLA shared epitope and with a worse functional and radiological outcome; and a population without ACPAs but with reactivities against several human cartilage gp39 peptides and type II collagen, with no increased prevalence of the HLA shared epitope and with a better radiological and functional prognosis. Also, ACPA positivity, if observed in non-RA patients, can preferentially be observed in patients who carry the HLA shared epitope, suggesting an important association between ACPA and the HLA shared epitope [29].

## Conclusion

Detection of autoantibodies against human citrullinated fibrinogen performs as well as the anti-CCP2 ELISA, because it has similar diagnostic characteristics. Despite the similar diagnostic characteristics of the different ACPA tests, we found that the agreement between the different available assays is only moderate, especially in non-RA patients.

## Abbreviations

ACPA = anti-citrullinated protein/peptide antibody; ACR = American College of Rheumatology; AhFibA = anti-human fibrinogen (auto)antibodies; CCP = cyclic citrullinated peptide; ELISA = enzyme-linked immunosorbent assay; HRP = horseradish peroxidase; OD = optical density; PBS = phosphate-buffered saline; RA = rheumatoid arthritis; RF = rheumatoid factor; ROC = receiver operating curve.

## Competing interests

The authors declare that they have no competing interests.

## Authors' contributions

BVC and TC drafted the manuscript. BVC performed the statistical analysis. BVC, TC, LDR and AD constructed the datasets. BVC, TC, DD, DE, GS and FDK participated in the study design. LN, CC, MS, CV and GS participated in the development of the AhFibA ELISA. All authors read and approved the final manuscript.

## Figures and Tables

**Table 1:** Sensitivities and specificities of AhFibA and anti-CCP2 assay in the two populations

	Cut-off	Population 1	Population 2	
		
		Specificity	Sensitivity	Sensitivity
AhFibA antibody (OD)	0.027	0.945	0.685	0.800
	0.11	0.985	0.609	0.711
	0.12	0.987	0.609	0.700
	0.19	0.990	0.598	0.678
anti-CCP2 antibody (U/ml)	14.5	0.950	0.728	0.744
	37	0.985	0.674	0.680
	42	0.985	0.641	0.650
	135	0.990	0.565	0.551
RF	160	0.948	0.391	0.556
	640	0.985	0.163	0.244
	1280	0.993	0.087	0.122

AhFibA, anti-human fibrinogen (auto)antibodies; CCP, cyclic citrullinated peptide; OD, optical density; RF, rheumatoid factor.

**Table 2:** Agreement between AhFibA assay and anti-CCP2 assay at the 98.5% specificity level in population 1

			Anti-CCP2		Total	κ	
						
			Neg	Pos			
Non-RA	AhFibA	Neg	452	4	456	0.420	0.845
		Pos	4	3	7		
	Total		456	7	463		

RA	AhFibA	Neg	28	8	36	0.765	
		Pos	2	54	56		
	Total		30	62	92		

AhFibA, anti-human fibrinogen (auto)antibodies; CCP, cyclic citrullinated peptide; Neg, negative; Pos, positive; RA, rheumatoid arthritis.

**Table 3:** Sensitivities of the different ACPA assays in population 2 after dichotomization at specificity level ≥ 98%

	Sensitivity	Cut-off (at 98% specificity)	Ref.
AhFibA	70%	0.12 OD	[18]
Anti-CCP1	51%	92 U/ml	[11]
Anti-CCP2	65%	42 U/ml	[14]
Anti-PepA	57%	1	[13,14]
Anti-PepB	55%	1	[13,14]

ACPA, anti-citrullinated protein/peptide antibody; AhFibA, anti-human fibrinogen (auto)antibodies; CCP, cyclic citrullinated peptide; OD, optical density.

**Table 4:** Value of κ statistic between the different ACPA assays in population 2

	κ				
	AhFibA	Anti-CCP1	Anti-CCP2	Anti-PepA	Anti-PepB
AhFibA	-				
Anti-CCP1	0.552	-			
Anti-CCP2	0.710	0.609	-		
Anti-PepA	0.540	0.766	0.605	-	
Anti-PepB	0.618	0.811	0.679	0.842	-

Values for κ statistic were calculated after dichotomization with previously defined >98% specific cut-offs [5,14,18]. ACPA, anti-citrullinated protein/peptide antibody; AhFibA, anti-human fibrinogen (auto)antibodies; CCP, cyclic citrullinated peptide.
